# Dissecting the Association of Genetically Predicted Neuroticism with Coronary Artery Disease: A Two-Sample Mendelian Randomization Study

**DOI:** 10.3390/jpm12020288

**Published:** 2022-02-16

**Authors:** Tao Yan, Shijie Zhu, Changming Xie, Xingyu Chen, Miao Zhu, Fan Weng, Chunsheng Wang, Changfa Guo

**Affiliations:** 1 Department of Cardiovascular Surgery, Zhongshan Hospital, Fudan University, Shanghai 200032, China; 19211210038@fudan.edu.cn (T.Y.); zhu.shijie@zs-hospital.sh.cn (S.Z.); 20211210111@fudan.edu.cn (M.Z.); 21211210056@m.fudan.edu.cn (F.W.); wangchunsheng@fudan.edu.cn (C.W.); 2 Department of Cardiology, The Eighth Affiliated Hospital, Sun Yat-sen University, Shenzhen 518033, China; xiechm3@mail2.sysu.edu.cn; 3 Department of Psychiatry, Fifth Affiliated Hospital, Sun Yat-sen University, Zhuhai 519000, China; chenxy48@mail2.sysu.edu.cn

**Keywords:** neuroticism, coronary artery disease, Mendelian randomization

## Abstract

Background: Observational studies on the association between neuroticism and coronary artery disease (CAD) are still rare, and the results of existing studies are not consistent. The present study aimed to explore causal associations of neuroticism with CAD. Methods: The summary-level data of GWAS for neuroticism and 12 items used to assess neuroticism were extracted from the UK Biobank, and included up to 380,506 participants. The general data for CAD were obtained from the CARDIoGRAMplusC4D consortium, which assembled 60,801 CAD patients and 123,504 non-cases. Single-nucleotide polymorphisms associated with neuroticism and 12 items at genome-wide significance were explored as instrumental variables. Two-sample Mendelian randomization (TSMR) analyses were performed to evaluate causal associations amongst the genetically predicted neuroticism and 12 items with CAD. Results: The present TSMR study did not reveal the genetic association of neuroticism with CAD. The calculated ORs for CAD using inverse-variance weighted, weighted median, and MR-Egger analysis were 1.12 (*p*-value = 0.187), 0.99 (*p*-value = 0.943), and 0.82 (*p*-value = 0.683), respectively. Further TSMR analysis of 12 dichotomous items for assessing neuroticism suggested that mood swings genetically increased the risk of CAD (OR = 1.67, *p*-value < 0.001). Conclusions: This study reported no genetically causal association of neuroticism with CAD. The present study also found that mood swings may genetically increase the risk of CAD. These findings may highlight the potential of mood control as a preventive measure for CAD.

## 1. Introduction

Neuroticism involves a pattern of irritability, anger, sadness, anxiety, worry, hostility, self-consciousness, and vulnerability [1,2], which reflects the process of individual emotion regulation and the tendency to experience negative emotions and emotional instability, which has far-reaching implications for public health [3]. Highly neurotic individuals tend to have psychological pressure, unrealistic thoughts, excessive demands, and impulses [4]. Previous studies have indicated that neuroticism is significantly associated with various Axis I and II mental disorders [5,6,7]. Moreover, increasing evidence has shown correlations between neuroticism and physical problems, such as cardiovascular diseases [8,9], cancers, [10,11] and allergic diseases [12].

Coronary artery disease (CAD) is one of the most common cardiovascular diseases in the clinic, greatly increasing the burden on both patients and public health [13]. Patients with CAD need to regularly take drugs to stabilize atherosclerotic plaques, and patients with severe cases even need to receive percutaneous coronary intervention or coronary-artery-bypass grafting treatment, which significantly reduces the quality of life of patients [14]. The occurrence and development of CAD are associated with the interplay of diverse factors, including genetic, psychosocial, and environmental factors [15,16]. However, research on the association between neuroticism and CAD is still relatively rare, and the results of existing studies are not consistent. Several investigations have suggested that neuroticism is related to increased angina-like complaints, but is not causally or etiologically related to CAD [17,18]. On the contrary, a recent meta-analysis indicated that some neuroticism patterns, such as anxiety and depression, were identified as etiologic and prognostic factors in CAD patients [19,20]. In fact, observational studies often contain potentially unmeasured information in terms of confounders and reverse causality, which makes it difficult to assess causal associations between neuroticism and CAD. We need more evidence that is not affected by potential confounding factors to explore the causal effect of neuroticism on CAD.

Mendelian randomization (MR) is an alternative method for potential causal inference [21], and uses single-nucleotide polymorphisms (SNPs) as instrumental variables of exposure factors to estimate the causal relationship between exposures and outcomes [22]. Due to the random allocation of alleles for a particular SNP, genetic variation will not be affected by potential confounding factors. In addition, genetic variation is established just before the onset of disease, which can avoid the possibility of a reverse-causal association.

In the present study, given the uncertainties about the causal role of neuroticism for CAD, we aimed to evaluate the potential causal association of neuroticism with CAD using two-sample Mendelian randomization (TSMR) analysis.

## 2. Materials and Methods

### 2.1. Data Sources

The Eysenck Personality Questionnaire, Revised Short Form (EPQ-R-S) [23], which included 12 dichotomous items (Table 1), was utilized to evaluate neuroticism. Individuals completing less than nine items were excluded from further analysis. The summary-level GWAS data for neuroticism and 12 items were acquired from the previous meta-analysis of two separate samples released in two different phases (May 2015 and July 2017) of the UK Biobank Study [24]. For CAD, we drew on aggregated statistics from the Coronary ARtery DIsease Genome-wide Replication and Meta-analysis plus The Coronary Artery Disease Genetics (CARDIoGRAMplusC4D) consortium, which assembled 60,801 CAD patients and 123,504 non-cases from 48 studies for a GWAS meta-analysis [25]. Due to the present study being a re-analysis of published data, no ethics approval was required.

### 2.2. Statistical Analysis

SNPs associated with neuroticism at genome-wide significance (*p*-value < 5 × 10^−8^) were explored as instrumental variables, which were then extracted from GWAS data for CAD. If a particular instrumental variable requested was not available in the CAD GWAS, a proxy SNP was searched instead using the 1000 Genomes European reference population. The two-sample Mendelian randomization analysis was conducted using the “TwoSampleMR” package in R language (version 4.1.2). The inverse-variance weighted (IVW) approach was utilized to evaluate the causal association between exposure and outcome through weighting and summing the influence of each instrumental variable, assuming that all the instruments are valid [26]. Since the IVW method limits the intercept to zero, the results may be biased if the instrument SNPs show horizontal pleiotropy. To strengthen the robustness of our findings, the weighted-median approach and MR-Egger approach were also performed, although the cost is reduced statistical power. The weighted-median approach allows, at most, 50% of the instruments weighting on the exposure to be invalid [27]. The MR-Egger approach is implemented through a simple modification of the weighted linear regression of IVW, that is, the intercept is not constrained to zero, but is estimated as part of the analysis [28]. The intercept can be used to assess a horizontal pleiotropic pathway. If the intercept of MR-Egger regression analysis has a *p*-value > 0.05, it indicates that no horizontal pleiotropic pathway exists. The leave-one-out sensitivity analysis, leaving out each SNP in turn, was then utilized to determine the impact of a single SNP on the analysis [29].

## 3. Results

### 3.1. Findings for Neuroticism on CAD

**Figure 1:**
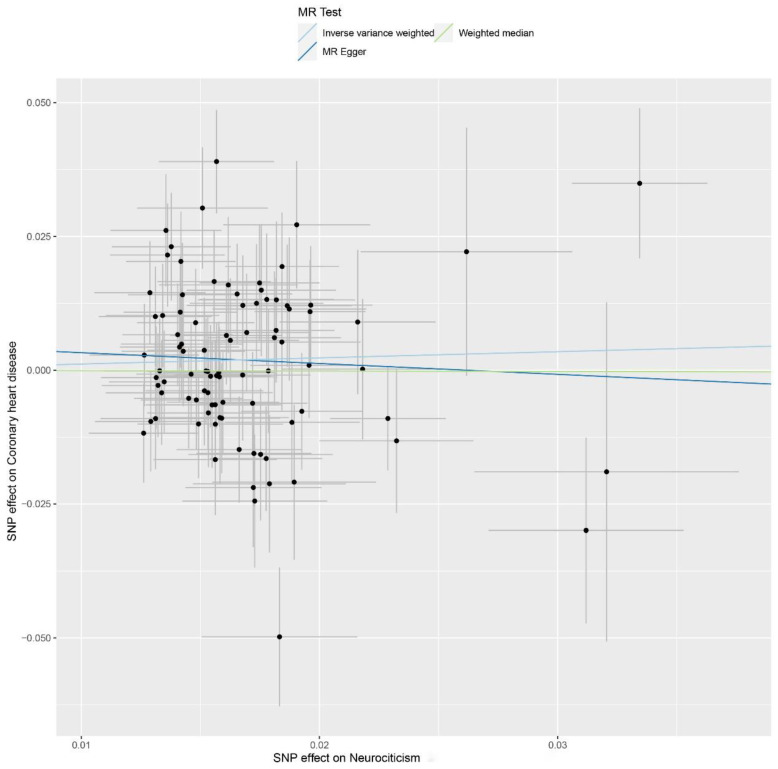
Genetic associations between neuroticism (exposure) and coronary artery disease (outcome).

**Figure 2:**
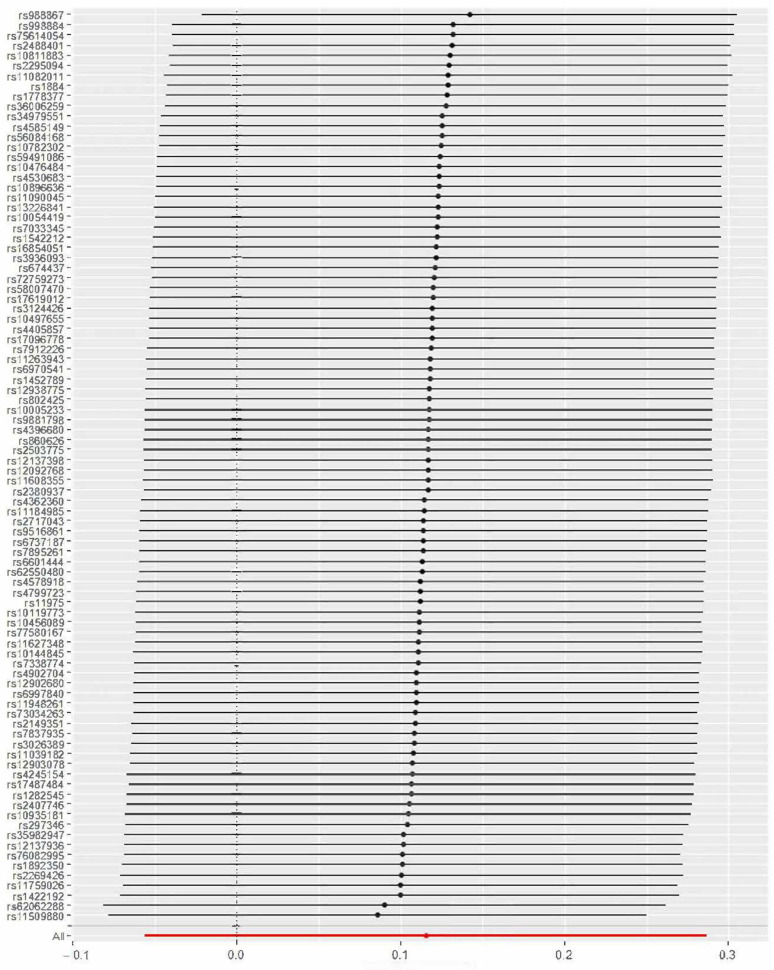
The leave-one-out plot for Mendelian randomization of neuroticism on coronary disease.

A total of 96 instrumental variables were identified, details of which are shown in Table S1. The calculated ORs (95% CIs) for CAD using IVW, weighted median, and MR-Egger analysis were 1.12 (0.95, 1.33; *p*-value = 0.187), 0.99 (0.80, 1.23; *p*-value = 0.943), and 0.82 (0.31, 2.15; *p*-value = 0.683), respectively. The TSMR estimate was not statistically significant, which revealed no evidence of the causal effect of neuroticism on CAD genetically (Figure 1). The intercept term from MR-Egger regression analysis (0.0053, *p*-value = 0.515) indicated that no horizontal pleiotropic pathway existed in the analysis. Moreover, the leave-one-out sensitivity analysis demonstrated that no single SNP drove these results (Figure 2).

### 3.2. Findings for 12 Dichotomous Items of EPQ-R-S on CAD

Instrumental variables for 12 dichotomous items are presented in Tables S2–S13. We found that genetically predicted “experiencing mood swings” significantly increased the risk of incident CAD. The left 11 items, however, showed no significant causal effects on CAD (Table 2). The intercept term of the MR-Egger regression indicated that “Hurt” (*p*-value = 0.0368) and “Irritableness” (*p*-value = 0.0267) have genetic pleiotropy, and the causal estimate may be biased due to the influence of the horizontal pleiotropic pathway. The other 10 items showed no horizontal pleiotropy (Table 3).

## 4. Discussion

In the present study, we conducted a TSMR analysis assessing the causal effect of neuroticism in the development of CAD. In line with several previous observational studies [17,18], no association of genetically predicted neuroticism on CAD was observed. Afterwards, we performed TSMR analysis to evaluate associations between 12 dichotomous items of EPQ-R-S and CAD, respectively. A novel finding is that experiencing mood swings significantly increased the risk of incident CAD, which requires replication in future studies.

Neuroticism is manifested as a negative emotional tendency, which describes the stable individual differences in experiencing negative emotions [30]. Growing evidence has suggested correlations between neuroticism and cardiovascular disorders. Previous studies have indicated that neuroticism is related to symptoms of CAD, such as chest pain or anginal symptoms [17,18], whereas the results of coronary angiography demonstrated that neuroticism was not associated with the pathophysiologic evidence of CAD. However, findings are inconsistent. Some recent prospective clinical studies have suggested that neuroticism increased the risk of CAD and mortality, compared to the ordinary population [31,32]. Although Kristin Torgersen et al. identified polygenic overlap between neuroticism and CAD [33], indicating that genetic factors may partly cause the comorbidity, our robust TSMR analysis showed no causal association between genetically predicted neuroticism and CAD. That neuroticism did not show significant causal effects on CAD should be interpreted with caution. Considering that high neuroticism may lead to unhealthy behaviors, namely smoking and sleep disorders, which are also risk factors for CAD [34,35,36], we suppose that those unhealthy behaviors are the causal link between neuroticism and CAD. However, from a more fundamental point of view—that is, from a genetic viewpoint—neuroticism does not increase the risk of CAD. The combined effect of psychological influence and unhealthy behaviors may explain part of the influence of neuroticism on CAD. Reducing these unhealthy behaviors may be beneficial to the prevention of CAD for individuals with neuroticism.

On the other hand, when focusing on a more detailed pattern of neuroticism, previous studies have shown that anxiety and depression were identified as etiologic and prognostic factors in CAD patients [19,20]. We further performed TSMR analysis based on the 12 dichotomous items of EPQ-R-S for evaluating neuroticism to assess the impact of each individual item on the risk of CAD. Only the genetically predicted “experiencing mood swings” significantly increased the risk of incident of CAD. Our results indicated that controlling mood swings may help reduce the risk of CAD. Contrary to our finding, a previous study in 868 menopausal women showed that mood swings were not associated with CAD [37]. However, the sample size of this study was too small and was limited to menopausal women. More large-scale observational studies or randomized clinical trials need to be conducted to verify our findings.

### Strengths of the Research and Its Limitations

The present study has several strengths. First, TSMR is an alternative method for potential causal inference. The association between genotype and outcome can represent the effect of the exposure on the outcome. Since alleles follow the principle of random allocation, genetic variation will not be affected by potential confounding factors. In addition, genetic variation is established just before the onset of disease, which can avoid the possibility of a reverse causal association. The utilization of TSMR analysis in the present study could reduce the potential confounding and reverse causality in observational research. Second, we included a large-scale population in the present study. Summary-level data of GWAS for neuroticism and 12 items were used to assess neuroticism and were extracted from the UK Biobank, and included up to 380,506 participants. The general data for CAD were obtained from the CARDIoGRAMplusC4D consortium, which assembled 60,801 CAD patients and 123,504 non-cases. The large study samples allowed our statistical analyses to have high power.

There are also several limitations in our study. First, although we determined instrumental variables of neuroticism and 12 dichotomous items of EPQ-R-S used for MR analysis through the huge sample size of the UK Biobank, it is impossible to completely rule out the possibility that SNPs related to neuroticism affect the risk of CAD through other causal pathways rather than neuroticism exposure, which is known as horizontal pleiotropy. In the present study, we conducted sensitivity analyses to eliminate horizontal pleiotropy as much as possible. Our sensitivity analysis results showed strong robustness, indicating that the bias from horizontal pleiotropy could almost be ignored. Second, since what we have obtained are summary-level data, it is impossible to conduct a more detailed stratified analysis of CAD patients. Third, our study mainly included individuals of European ancestry, and thus, cannot be generalized to other ethnicities. More analyses need to be conducted to assess the reliability of our findings in other ancestral populations.

Despite these limitations, we provided novel insights into causal associations between neuroticism and CAD using TSMR analysis. Our research reduced the unmeasured confounding factors and reverse causality that may exist in observational analysis. We found that mood swings were associated with an increased risk of CAD. We can conduct some large-scale observational studies or prospective studies in the future to verify the results, based on the present study. Furthermore, conducting more in-depth studies of molecular mechanisms may help us better understand the relationship between mood swings and CAD. If controlling mood swings can really reduce the risk of suffering from CAD, it will greatly reduce medical burdens.

## 5. Conclusions

In conclusion, the present study evaluated causal associations of neuroticism with CAD using TSMR analysis. Our findings showed no causal effect of neuroticism on CAD, whereas mood swings were associated with an increased risk of CAD. Controlling mood swings may help to reduce the risk of CAD.

## Figures and Tables

**Table 1:** Content and abbreviations of the 12 Eysenck Personality Questionnaire, Revised Short-Form neuroticism items.

Abbreviations	Item
Irritableness	Are you an irritable person?
Loneliness	Do you often feel lonely?
Misery	Do you ever feel ‘just miserable’ for no reason?
Mood swings	Does your mood often go up and down?
Feeling fed up	Do you often feel ‘fed up’?
Feeling nervous	Would you call yourself a nervous person?
Worrier	Are you a worrier?
Feeling tense	Would you call yourself tense or ‘highly strung’?
Suffering from nerves	Do you suffer from ‘nerves’?
Hurt	Are your feelings easily hurt?
Worrying after embarrassment	Do you worry too long after an embarrassing experience?
Guilt	Are you often troubled by feelings of guilt?

**Table 2:** Association of genetically predicted 12 items with CAD.

Item	Method	OR (95% CI)	*p*-Value
Mood swings	IVW	1.67 (1.27, 2.19)	<0.001
	Weighted median	1.60 (1.15, 2.22)	0.00519
Feeling fed up	IVW	1.47 (0.99, 2.18)	0.0593
	Weighted median	1.41 (0.95, 2.09)	0.0893
Guilt	IVW	1.21 (0.66, 2.23)	0.539
	Weighted median	1.44 (0.84, 2.47)	0.190
Hurt	IVW	0.79 (0.55, 1.12)	0.180
	Weighted median	0.91 (0.63, 1.33)	0.638
Loneliness	IVW	1.26 (0.73, 2.18)	0.412
	Weighted median	1.43 (0.75, 2.71)	0.277
Misery	IVW	1.15 (0.84, 1.58)	0.387
	Weighted median	1.06 (0.74, 1.51)	0.750
Feeling nervous	IVW	1.23 (0.87, 1.73)	0.250
	Weighted median	1.36 (0.96, 1.93)	0.0823
Feeling tense	IVW	1.21 (0.77, 1.90)	0.409
	Weighted median	0.82 (0.54, 1.23)	0.339
Worrier	IVW	1.07 (0.77, 1.48)	0.704
	Weighted median	1.04 (0.75, 1.43)	0.821
Irritableness	IVW	1.14 (0.87, 1.48)	0.343
	Weighted median	1.19 (0.86, 1.65)	0.285
Suffering from nerves	IVW	1.00 (1.00, 1.00)	0.830
	Weighted median	1.00 (1.00, 1.00)	0.0846
Worrying after embarrassment	IVW	0.84 (0.55, 1.28)	0.429
	Weighted median	0.73 (0.48, 1.12)	0.146

CAD: coronary artery disease; IVW: inverse-variance weighted.

**Table 3:** Intercepts of MR-Egger regression analysis of 12 items.

Item	Intercepts	*p*-Value
Mood swings	−0.0227	0.0959
Feeling fed up	−0.0351	0.842
Guilt	−0.0355	0.336
Hurt	−0.0266	0.0368
Loneliness	−0.00791	0.794
Misery	−0.0207	0.0649
Feeling nervous	−0.0138	0.402
Feeling tense	−0.00923	0.746
Worrier	0.00833	0.667
Irritableness	−0.0354	0.0267
Suffering from nerves	−0.0368	0.546
Worrying after embarrassment	−0.00324	0.877

## Data Availability

The data presented in this study are available in the public database The MRC IEU OpenGWAS data infrastructure (https://gwas.mrcieu.ac.uk/).

## Supplementary Materials

The following supporting information can be downloaded at: https://www.mdpi.com/article/10.3390/jpm12020288/s1, Table S1: Instrumental variables for neuroticism; Table S2: Instrumental variables for “Mood swings”; Table S3: Instrumental variables for “Feeling fed-up”; Table S4: Instrumental variables for “Guilt”; Table S5: Instrumental variables for “Hurt”; Table S6: Instrumental variables for “Loneliness”; Table S7: Instrumental variables for “Misery”; Table S8: Instrumental variables for “Feeling nervous”; Table S9: Instrumental variables for “Feeling tense”; Table S10: Instrumental variables for “Worrier”; Table S11: Instrumental variables for “Irritableness”; Table S12: Instrumental variables for “Suffering from nerves”; Table S13: Instrumental variables for “Worrying after embarrassment”.
