# Supplementary material for: Dissecting the Association of Genetically Predicted Neuroticism with Coronary Artery Disease: A Two-Sample Mendelian Randomization Study
Source: J Pers Med. 2022 Feb 16;12(2):288. doi: 10.3390/jpm12020288 (PMC8877774; doi:10.3390/jpm12020288)
Supplement: Supplementary file 1 [file jpm-12-00288-s001.zip › jpm-1576041-supplementary.pdf]

Table S1. Instrumental variables for neuroticism.

No.	SNP	Effect allele	Other allele	Beta	Standard error	*p*-value	Eaf
1	rs12137398	T	C	0.0178531	0.00292386	1.02E-09	0.19435
2	rs2488401	T	C	-0.0172249	0.00285795	1.67E-09	0.209268
3	rs11263943	G	A	-0.0157982	0.00232943	1.19E-11	0.571173
4	rs17096778	G	A	-0.0320548	0.00554869	7.60E-09	0.0452785
5	rs11184985	C	T	-0.0142811	0.00258668	3.37E-08	0.269475
6	rs12137936	G	C	0.0137782	0.00248705	3.03E-08	0.307137
7	rs12092768	A	G	-0.0153096	0.00254524	1.79E-09	0.284798
8	rs4396680	G	A	-0.0167747	0.00285091	4.00E-09	0.796901
9	rs2717043	T	C	0.0151639	0.00238015	1.87E-10	0.627326
10	rs988867	T	G	0.0183242	0.00326519	2.00E-08	0.855723
11	rs998884	G	A	0.0177656	0.00235994	5.16E-14	0.401456
12	rs59491086	G	A	-0.0188454	0.00284072	3.27E-11	0.205198
13	rs17619012	T	G	-0.0156225	0.00279422	2.26E-08	0.216843
14	rs6737187	A	G	-0.0141254	0.00250405	1.69E-08	0.299488
15	rs10497655	C	T	-0.0151713	0.0024705	8.19E-10	0.314337
16	rs9881798	C	A	0.015771	0.0023406	1.60E-11	0.410499
17	rs1282545	C	T	0.0167823	0.00234816	8.84E-13	0.436332
18	rs10935181	A	G	-0.0165427	0.00232243	1.06E-12	0.424096
19	rs1542212	G	T	0.0159476	0.00236156	1.45E-11	0.39305
20	rs4405857	C	G	0.0133764	0.00234673	1.20E-08	0.598404
21	rs4585149	C	T	0.0175304	0.00302927	7.16E-09	0.822931
22	rs16854051	T	C	-0.0156286	0.00283847	3.66E-08	0.213166
23	rs10005233	T	C	0.0146127	0.0023023	2.19E-10	0.521594
24	rs4530683	G	A	0.0129193	0.00234131	3.43E-08	0.585044
25	rs1422192	A	G	0.0190464	0.00308544	6.68E-10	0.165603
26	rs58007470	T	C	0.0154904	0.00279509	2.99E-08	0.218522
27	rs10476484	G	A	0.0149229	0.00256362	5.85E-09	0.276744
28	rs11948261	A	T	0.0177896	0.00282464	3.01E-10	0.210714
29	rs77580167	T	G	-0.018192	0.00330402	3.68E-08	0.141046
30	rs10054419	G	T	-0.0131218	0.00231302	1.40E-08	0.43857
31	rs34979551	G	A	-0.0189387	0.00343466	3.50E-08	0.127858
32	rs2503775	G	A	-0.021811	0.0034	1.41E-10	0.868869
33	rs28986304	C	T	0.0212038	0.00345114	8.05E-10	0.126444
34	rs11759026	G	A	-0.0150881	0.0027408	3.68E-08	0.229136
35	rs10456089	A	G	-0.0261729	0.00444286	3.83E-09	0.0758129
36	rs2269426	A	G	0.0184312	0.00239025	1.25E-14	0.359398
37	rs10782302	C	T	0.0126181	0.0022988	4.05E-08	0.497033
38	rs2056477	C	G	-0.0156998	0.00272471	8.32E-09	0.231276
39	rs802425	T	C	-0.0131476	0.00231472	1.34E-08	0.439978
40	rs13226841	C	T	0.017193	0.00229669	7.13E-14	0.476138

41	rs6970541	G	T	-0.0134835	0.00231159	5.45E-09	0.50864
42	rs1731951	A	T	-0.0136621	0.00234946	6.07E-09	0.439961
43	rs11509880	A	G	0.0156797	0.00241822	8.92E-11	0.344232
44	rs7837935	G	T	0.0175613	0.00313315	2.08E-08	0.840353
45	rs6997840	C	T	0.0131099	0.00237197	3.25E-08	0.606911
46	rs2407746	G	C	0.0174863	0.00251638	3.68E-12	0.301794
47	rs6601444	T	C	0.0162582	0.00289394	1.94E-08	0.200027
48	rs75614054	T	C	0.0311936	0.00409312	2.52E-14	0.0864162
49	rs62550480	T	C	-0.0181085	0.00279539	9.32E-11	0.214237
50	rs10811883	T	C	-0.01725	0.00241597	9.32E-13	0.626255
51	rs72759273	G	A	-0.0158225	0.00289789	4.76E-08	0.194462
52	rs2380937	C	T	-0.0132918	0.00234962	1.54E-08	0.399161
53	rs11975	C	A	0.0216038	0.0032738	4.14E-11	0.144253
54	rs10119773	G	A	0.0160955	0.00235555	8.33E-12	0.534002
55	rs7033345	C	T	-0.0153362	0.00256931	2.38E-09	0.27906
56	rs2149351	G	T	-0.0186461	0.00270194	5.15E-12	0.761015
57	rs7895261	G	T	-0.0142086	0.00254361	2.33E-08	0.28996
58	rs7912226	A	T	-0.0132233	0.00236341	2.20E-08	0.400911
59	rs860626	G	T	-0.0152341	0.00254198	2.06E-09	0.297635
60	rs297346	G	A	-0.0155813	0.00238611	6.56E-11	0.633883
61	rs1884	C	G	0.0166294	0.00262749	2.46E-10	0.256742
62	rs4757136	A	T	-0.0166419	0.00232689	8.56E-13	0.571192
63	rs73034263	G	C	-0.0161736	0.00291153	2.78E-08	0.194034
64	rs674437	A	G	-0.0145107	0.00229854	2.74E-10	0.514943
65	rs10896636	G	C	0.0158966	0.00244977	8.62E-11	0.336471
66	rs4245154	G	A	-0.0196045	0.00232833	3.75E-17	0.431123
67	rs3026389	G	C	-0.017359	0.00281117	6.60E-10	0.788882
68	rs11039182	C	T	0.0196363	0.00258543	3.08E-14	0.269312
69	rs11608355	C	T	0.019565	0.00247157	2.44E-15	0.316757
70	rs9516861	A	T	0.0184203	0.00330409	2.48E-08	0.141405
71	rs3124426	T	C	-0.0153159	0.00272379	1.88E-08	0.769532
72	rs7338774	G	A	0.0148001	0.00246217	1.85E-09	0.329767
73	rs1892350	G	A	0.0141816	0.00229997	7.00E-10	0.490613
74	rs4902704	C	G	0.0141562	0.00237123	2.38E-09	0.623827
75	rs36006259	T	C	-0.0179004	0.00320796	2.41E-08	0.157665
76	rs1778377	T	A	0.015626	0.00258666	1.53E-09	0.728832
77	rs11627348	A	C	0.0187324	0.0032275	6.48E-09	0.151394
78	rs10144845	T	C	0.0181806	0.00245849	1.41E-13	0.679702
79	rs12902680	C	T	0.0134144	0.00238139	1.77E-08	0.381068
80	rs4362360	C	T	-0.0126458	0.00229465	3.57E-08	0.487107
81	rs3936093	G	A	0.0148324	0.00233104	1.98E-10	0.567092
82	rs12903078	A	G	0.0128833	0.00233731	3.54E-08	0.406166
83	rs62055866	C	G	0.0148339	0.0023587	3.19E-10	0.614957
84	rs3785237	C	G	0.0166058	0.00231311	7.03E-13	0.503161

85	rs76082995	C	T	0.0135575	0.00232867	5.81E-09	0.437994
86	rs12938775	A	G	-0.0156852	0.00229384	8.00E-12	0.500314
87	rs35982947	C	A	-0.0136214	0.00240703	1.52E-08	0.368951
88	rs62062288	A	G	0.0334444	0.00284246	5.82E-32	0.211505
89	rs56084168	T	C	-0.0232368	0.00323453	6.78E-13	0.147994
90	rs1452789	A	T	-0.0154286	0.00236599	6.98E-11	0.618407
91	rs4799723	G	T	0.0140485	0.00231594	1.31E-09	0.52019
92	rs11082011	T	C	-0.0228736	0.00242768	4.44E-21	0.656005
93	rs17487484	G	T	0.0142497	0.0022939	5.24E-10	0.50301
94	rs4578918	C	T	-0.0169464	0.00263183	1.20E-10	0.743183
95	rs2295094	A	G	0.0172829	0.00303849	1.28E-08	0.174406
96	rs11090045	A	G	0.0192599	0.0025466	3.93E-14	0.30231

Table S2. Instrumental variables for “Mood swings”.

No.	SNP	Effect allele	Other allele	Beta	Standard error	*p*-value	Eaf
1	rs4651205	T	C	-0.01421	0.002586	3.91E-08	0.28022
2	rs11184994	T	C	0.013925	0.002508	2.82E-08	0.691491
3	rs12137936	G	C	0.015202	0.002509	1.38E-09	0.307137
4	rs35789697	A	G	-0.01437	0.002418	2.82E-09	0.358174
5	rs11687833	T	C	0.013512	0.00241	2.06E-08	0.3941
6	rs2678897	A	G	0.014176	0.002393	3.12E-09	0.611438
7	rs34759087	T	C	-0.0212	0.00359	3.53E-09	0.117824
8	rs9852417	A	C	-0.01327	0.002378	2.42E-08	0.598536
9	rs600011	C	A	0.0149	0.002554	5.38E-09	0.29221
10	rs6895295	T	C	0.01522	0.002789	4.86E-08	0.223658
11	rs68152875	A	C	-0.0137	0.00238	8.61E-09	0.610068
12	rs6889822	G	A	-0.01395	0.002381	4.66E-09	0.385749
13	rs9344688	G	A	-0.01404	0.002443	8.97E-09	0.351818
14	rs1890408	T	C	-0.01953	0.003548	3.70E-08	0.878874
15	rs12524535	G	A	0.026334	0.004566	8.06E-09	0.069011
16	rs67447472	T	G	0.024587	0.003807	1.06E-10	0.104156
17	rs11509880	A	G	0.016099	0.00244	4.18E-11	0.344232
18	rs1833070	A	G	-0.01337	0.002438	4.20E-08	0.361165
19	rs1962104	C	T	0.015168	0.002363	1.38E-10	0.550375
20	rs10983783	T	G	-0.01332	0.002328	1.04E-08	0.553347
21	rs56318386	T	C	-0.01713	0.002827	1.37E-09	0.215156
22	rs4836789	C	T	0.013486	0.002363	1.15E-08	0.597128
23	rs1360379	T	C	-0.01436	0.002367	1.31E-09	0.604417
24	rs7895261	G	T	-0.01413	0.002567	3.70E-08	0.28996
25	rs11039149	G	A	0.017658	0.002608	1.29E-11	0.269477
26	rs297346	G	A	-0.01501	0.002408	4.58E-10	0.633883
27	rs12420205	T	C	0.016451	0.002501	4.77E-11	0.677505
28	rs28655666	A	G	-0.01441	0.002324	5.66E-10	0.544253
29	rs1373921	G	A	0.019478	0.00275	1.41E-12	0.230326
30	rs9671386	A	G	0.015851	0.002651	2.24E-09	0.743836
31	rs56059702	T	G	-0.01535	0.00233	4.46E-11	0.556652
32	rs7202252	C	T	0.014328	0.002624	4.75E-08	0.733432
33	rs9929242	A	G	-0.01298	0.002331	2.57E-08	0.528828
34	rs55657917	G	T	0.026183	0.002838	2.81E-20	0.213403
35	rs1788014	G	A	0.014718	0.002354	4.07E-10	0.443118
36	rs11082011	T	C	-0.01672	0.00245	8.74E-12	0.656005
37	rs56403421	C	A	0.018488	0.002493	1.21E-13	0.327025
38	rs784256	A	G	0.020839	0.002999	3.70E-12	0.815002
39	rs4578918	C	T	-0.01609	0.002656	1.38E-09	0.743183
40	rs11090039	A	G	0.016442	0.002576	1.75E-10	0.283315

Table S3. Instrumental variables for “Feeling fed-up”.

No.	SNP	Effect allele	Other allele	Beta	Standard error	*p*-value	Eaf
1	rs11209175	T	C	-0.01471	0.002404	9.43E-10	0.627539
2	rs34668726	G	C	0.02193	0.003123	2.19E-12	0.164731
3	rs17711053	G	A	-0.01881	0.003374	2.47E-08	0.136297
4	rs17194468	T	C	0.020798	0.003626	9.71E-09	0.115026
5	rs1439253	A	G	-0.01465	0.002343	4.07E-10	0.421921
6	rs9842349	T	C	-0.01422	0.002506	1.40E-08	0.307085
7	rs4625	G	A	-0.01615	0.002513	1.30E-10	0.303255
8	rs17018482	A	C	0.019131	0.002945	8.19E-11	0.191739
9	rs77087420	G	A	-0.02895	0.00519	2.41E-08	0.052365
10	rs997091	C	A	-0.01405	0.00241	5.56E-09	0.367352
11	rs6862766	T	C	-0.01436	0.002334	7.61E-10	0.553521
12	rs10044479	C	G	-0.01396	0.00234	2.40E-09	0.436241
13	rs2149351	G	T	-0.01495	0.002722	3.98E-08	0.761015
14	rs7912226	A	T	-0.01537	0.002381	1.06E-10	0.400911
15	rs10838635	A	G	0.016953	0.00269	2.96E-10	0.752836
16	rs7334060	C	T	-0.01319	0.002413	4.62E-08	0.357385
17	rs10144845	T	C	0.015147	0.002477	9.60E-10	0.679702
18	rs4899292	G	A	0.013678	0.002439	2.05E-08	0.659554
19	rs783537	G	A	-0.01429	0.002422	3.63E-09	0.648644
20	rs34641928	G	A	0.015862	0.002746	7.65E-09	0.2327
21	rs61744010	T	G	0.012822	0.002335	3.97E-08	0.437854
22	rs4630591	T	C	0.025029	0.002931	1.34E-17	0.198844
23	rs11877032	T	A	0.014159	0.002347	1.60E-09	0.423142
24	rs4799949	T	C	-0.01769	0.002435	3.77E-13	0.655361
25	rs599550	A	G	0.024833	0.003266	2.88E-14	0.852215
26	rs10409264	A	G	-0.0203	0.003071	3.90E-11	0.171851
27	rs6031966	T	G	0.013207	0.002316	1.18E-08	0.467987

Table S4. Instrumental variables for "Guilt".

No.	SNP	Effect allele	Other allele	Beta	Standard error	*p*-value	Eaf
1	rs681875	A	C	0.017006	0.002872	3.18E-09	0.206279
2	rs34657012	A	C	0.015477	0.002585	2.13E-09	0.279037
3	rs780024	A	T	0.013542	0.002473	4.37E-08	0.341371
4	rs56343114	G	C	0.024797	0.003301	5.83E-14	0.143745
5	rs12528131	G	A	0.014922	0.002324	1.36E-10	0.4826
6	rs6986	C	G	0.020144	0.00278	4.31E-13	0.223335
7	rs10119773	G	A	0.015735	0.002378	3.67E-11	0.534002
8	rs35623509	G	C	-0.01468	0.002614	1.97E-08	0.271529
9	rs12420205	T	C	0.014957	0.002502	2.26E-09	0.677505
10	rs55769038	A	G	-0.01608	0.002351	7.83E-12	0.578002
11	rs2109648	A	G	0.01411	0.002416	5.22E-09	0.63965
12	rs77804065	T	C	0.017506	0.002834	6.48E-10	0.217158
13	rs1557339	A	C	-0.01666	0.002659	3.69E-10	0.738067

Table S5. Instrumental variables for "Hurt".

No.	SNP	Effect allele	Other allele	Beta	Standard error	*p*-value	Eaf
1	rs2488401	T	C	-0.01865	0.00289	1.11E-10	0.209268
2	rs12028465	A	G	-0.01366	0.002369	8.05E-09	0.400161
3	rs4652676	A	G	0.014807	0.002701	4.17E-08	0.246938
4	rs219226	C	T	-0.01482	0.002718	4.97E-08	0.757344
5	rs10210652	A	G	0.015348	0.002438	3.08E-10	0.347252
6	rs1978573	T	C	-0.01428	0.002548	2.11E-08	0.300324
7	rs10511285	G	T	0.013083	0.002348	2.53E-08	0.459246
8	rs62268962	T	A	-0.01558	0.002821	3.35E-08	0.219591
9	rs4868774	G	A	-0.01727	0.003076	1.95E-08	0.177192
10	rs2027798	T	C	-0.01344	0.002362	1.28E-08	0.580897
11	rs8111	T	C	0.015318	0.002673	9.96E-09	0.251639
12	rs11767715	T	C	-0.02002	0.003447	6.29E-09	0.132418
13	rs34548930	G	C	0.019195	0.002322	1.36E-16	0.482404
14	rs17532098	T	C	0.021316	0.00383	2.63E-08	0.102583
15	rs145965565	G	T	0.026967	0.004052	2.82E-11	0.090646
16	rs10117184	G	A	-0.01603	0.002788	8.87E-09	0.231023
17	rs1231375	C	T	-0.01913	0.003362	1.26E-08	0.861237
18	rs73480560	T	C	0.015014	0.002682	2.16E-08	0.260712
19	rs10891564	A	G	0.014851	0.002381	4.48E-10	0.388119
20	rs10850379	T	C	0.014392	0.002346	8.51E-10	0.425983
21	rs2102923	G	A	0.013933	0.002414	7.79E-09	0.627608
22	rs4702	A	G	-0.01402	0.002338	2.00E-09	0.561859
23	rs12933611	G	T	0.013152	0.002409	4.75E-08	0.373652
24	rs62035176	A	G	-0.0134	0.002426	3.34E-08	0.641118
25	rs55657917	G	T	0.031728	0.002844	6.80E-29	0.213403
26	rs4791774	G	A	-0.01285	0.002341	4.07E-08	0.463908
27	rs11663050	G	T	-0.0197	0.002424	4.41E-16	0.642196
28	rs545853	A	G	-0.01307	0.002353	2.80E-08	0.537648
29	rs1389993	C	G	-0.01394	0.002367	3.85E-09	0.410538

Table S6. Instrumental variables for "Loneliness".

No.	SNP	Effect allele	Other allele	Beta	Standard error	*p*-value	Eaf
1	rs74338595	C	T	-0.01471	0.002562	9.31E-09	0.285559
2	rs4958586	A	G	0.013884	0.002384	5.72E-09	0.622638
3	rs12554512	C	T	-0.01282	0.002345	4.60E-08	0.409986
4	rs7044244	A	G	-0.01524	0.002395	1.95E-10	0.378475
5	rs11039389	C	T	-0.01452	0.002433	2.41E-09	0.341432
6	rs7209581	C	G	0.01298	0.002345	3.10E-08	0.433605
7	rs599550	A	G	0.021283	0.00326	6.60E-11	0.852215

Table S7. Instrumental variables for "Misery".

No.	SNP	Effect allele	Other allele	Beta	Standard error	*p*-value	Eaf
1	rs77417259	A	G	-0.04295	0.006813	2.89E-10	0.029615
2	rs3795310	T	C	0.014153	0.002318	1.02E-09	0.459361
3	rs12094143	G	C	-0.03567	0.006298	1.48E-08	0.034866
4	rs7534703	T	C	-0.04471	0.00815	4.12E-08	0.020596
5	rs2042555	A	G	0.013172	0.002349	2.05E-08	0.41045
6	rs72786276	A	G	0.019027	0.003435	3.04E-08	0.129617
7	rs13018407	C	T	0.013466	0.002435	3.18E-08	0.342545
8	rs2312147	C	T	0.017478	0.002382	2.15E-13	0.623954
9	rs114306779	T	G	0.030609	0.005558	3.65E-08	0.045464
10	rs9586	T	C	-0.01575	0.002785	1.57E-08	0.780036
11	rs67932684	G	A	-0.01381	0.002378	6.31E-09	0.390294
12	rs836927	A	C	0.013459	0.002364	1.24E-08	0.436326
13	rs45510091	G	A	-0.02957	0.005181	1.15E-08	0.05233
14	rs1422191	A	G	0.017326	0.003102	2.34E-08	0.165914
15	rs41286287	A	T	0.025766	0.004501	1.04E-08	0.070716
16	rs55893771	T	C	-0.0226	0.003285	5.94E-12	0.14549
17	rs1971655	T	C	0.01295	0.002314	2.19E-08	0.507905
18	rs10156548	C	G	-0.01519	0.002409	2.93E-10	0.62954
19	rs11599236	C	T	-0.01681	0.002384	1.75E-12	0.407423
20	rs7912226	A	T	-0.01314	0.002377	3.28E-08	0.400911
21	rs628246	C	T	-0.01678	0.003059	4.12E-08	0.828192
22	rs4245154	G	A	-0.01451	0.002342	5.88E-10	0.431123
23	rs11039149	G	A	0.019129	0.0026	1.87E-13	0.269477
24	rs4757142	A	G	-0.01372	0.002383	8.54E-09	0.596599
25	rs113829044	C	T	0.036444	0.006271	6.16E-09	0.035078
26	rs4424705	A	T	0.016047	0.002642	1.26E-09	0.256746
27	rs11608355	C	T	0.014502	0.002486	5.43E-09	0.316757
28	rs3936093	G	A	0.013658	0.002345	5.71E-09	0.567092
29	rs12945855	C	T	0.013403	0.002312	6.75E-09	0.467881
30	rs868150	G	A	-0.01362	0.002378	1.02E-08	0.589231
31	rs56280951	A	G	0.020238	0.002823	7.54E-13	0.21432
32	rs35994060	T	A	0.016006	0.002392	2.21E-11	0.387895
33	rs17681615	A	G	0.014544	0.002462	3.46E-09	0.329342
34	rs55731231	G	C	0.017674	0.002789	2.36E-10	0.221154
35	rs8097041	T	A	0.013225	0.002385	2.96E-08	0.624575

Table S8. Instrumental variables for “Feeling nervous”.

No.	SNP	Effect allele	Other allele	Beta	Standard error	*p*-value	Eaf
1	rs12729445	T	C	0.01382	0.002326	2.81E-09	0.540821
2	rs332828	A	G	0.013998	0.002331	1.93E-09	0.448949
3	rs10747488	A	C	0.015379	0.002746	2.13E-08	0.7661
4	rs13395141	T	C	0.012848	0.002357	4.97E-08	0.432417
5	rs1368549	T	G	0.017273	0.002323	1.05E-13	0.512188
6	rs10935184	C	T	-0.0138	0.002357	4.80E-09	0.407441
7	rs1375545	T	G	0.018904	0.002392	2.70E-15	0.615754
8	rs9835772	T	A	-0.0168	0.002727	7.26E-10	0.23748
9	rs9879090	C	T	-0.01404	0.002327	1.60E-09	0.54584
10	rs974711	A	G	0.014126	0.00233	1.34E-09	0.456529
11	rs9688806	T	C	0.022323	0.003501	1.81E-10	0.125717
12	rs12203592	T	C	0.015857	0.002844	2.46E-08	0.209969
13	rs707916	A	G	-0.01768	0.002405	1.94E-13	0.365563
14	rs6919397	G	T	-0.01387	0.002328	2.55E-09	0.459046
15	rs9267084	A	G	0.018268	0.003267	2.26E-08	0.148398
16	rs7792856	C	T	-0.01342	0.002371	1.51E-08	0.606287
17	rs6948180	A	G	0.014172	0.002324	1.07E-09	0.520942
18	rs2407746	G	C	0.015074	0.002541	2.99E-09	0.301794
19	rs4129585	C	A	-0.01501	0.002342	1.47E-10	0.573268
20	rs7026674	T	C	0.014594	0.002573	1.42E-08	0.288112
21	rs4919695	G	A	-0.0176	0.002372	1.20E-13	0.395999
22	rs2930456	C	G	0.01934	0.00351	3.59E-08	0.874798
23	rs11030107	G	A	-0.01579	0.002666	3.20E-09	0.252832
24	rs10501320	C	G	0.01624	0.002658	1.00E-09	0.25471
25	rs11619066	C	G	-0.01605	0.002819	1.25E-08	0.215297
26	rs2853779	A	G	-0.01615	0.002327	3.94E-12	0.54646
27	rs117618307	G	A	0.046743	0.008228	1.34E-08	0.02024
28	rs4787491	G	A	-0.01369	0.002325	3.90E-09	0.53475
29	rs8050237	G	T	-0.01593	0.002886	3.41E-08	0.796559
30	rs17884466	G	A	0.018942	0.003408	2.73E-08	0.133499
31	rs62062288	A	G	0.022911	0.002871	1.45E-15	0.211505
32	rs1427041	G	A	-0.01781	0.003044	4.94E-09	0.823726
33	rs62212171	C	T	0.021265	0.003369	2.75E-10	0.137405
34	rs2205129	A	G	-0.01422	0.002508	1.42E-08	0.316141
35	rs5758209	G	T	-0.01303	0.002325	2.09E-08	0.491215

Table S9. Instrumental variables for "Feeling tense".

No.	SNP	Effect allele	Other allele	Beta	Standard error	*p*-value	Eaf
1	rs10218528	A	T	-0.01321	0.002385	3.07E-08	0.400556
2	rs2100888	A	T	-0.01485	0.002417	8.05E-10	0.634351
3	rs4671330	A	G	0.015168	0.002459	6.93E-10	0.664616
4	rs79861172	A	G	0.021976	0.004022	4.65E-08	0.091816
5	rs2097247	T	C	0.023412	0.003945	2.94E-09	0.095825
6	rs9811546	A	G	-0.01461	0.002506	5.62E-09	0.311971
7	rs6883228	C	G	0.01653	0.003008	3.91E-08	0.183199
8	rs6891955	C	G	0.014538	0.002439	2.53E-09	0.354191
9	rs28738966	A	G	0.017533	0.002818	4.92E-10	0.217446
10	rs1147851	G	A	0.015105	0.002581	4.82E-09	0.284644
11	rs11509880	A	G	0.014884	0.002448	1.20E-09	0.344232
12	rs1450832	A	G	0.01566	0.002579	1.27E-09	0.290096
13	rs4129585	C	A	-0.01443	0.002347	7.92E-10	0.573268
14	rs3740393	C	G	-0.02008	0.003348	2.02E-09	0.140572
15	rs10767733	A	G	-0.01496	0.002386	3.61E-10	0.402825
16	rs4937872	G	A	0.015642	0.002381	5.04E-11	0.417009
17	rs9527336	A	G	-0.01529	0.002597	3.93E-09	0.722762
18	rs3751855	C	T	-0.01341	0.002376	1.68E-08	0.393888
19	rs7194615	C	T	0.013151	0.002367	2.75E-08	0.551498
20	rs56084168	T	C	-0.01789	0.003274	4.63E-08	0.147994
21	rs78379741	C	T	-0.01853	0.003012	7.63E-10	0.181714
22	rs11090045	A	G	0.016447	0.002578	1.77E-10	0.30231

Table S10. Instrumental variables for “Worrier”.

No.	SNP	Effect allele	Other allele	Beta	Standard error	*p*-value	Eaf
1	rs2488398	C	G	-0.02023	0.002825	8.00E-13	0.215362
2	rs2367724	T	C	0.01466	0.002463	2.63E-09	0.668756
3	rs6685497	A	T	0.013561	0.002349	7.85E-09	0.428177
4	rs7543687	T	C	-0.01643	0.002956	2.75E-08	0.201925
5	rs4659554	G	A	-0.01413	0.002572	3.98E-08	0.285395
6	rs7567451	T	G	0.014261	0.002587	3.52E-08	0.720293
7	rs4396680	G	A	-0.01631	0.00288	1.49E-08	0.796901
8	rs1375311	A	G	0.016509	0.002874	9.24E-09	0.207134
9	rs6798941	T	C	-0.01949	0.002563	2.85E-14	0.291124
10	rs62250713	G	A	0.020154	0.002396	4.08E-17	0.624582
11	rs1542212	G	T	0.013804	0.002386	7.20E-09	0.39305
12	rs57462170	A	G	0.022544	0.00374	1.66E-09	0.107943
13	rs1697692	T	C	0.013088	0.002337	2.15E-08	0.538058
14	rs6791142	C	T	0.014331	0.002383	1.81E-09	0.615571
15	rs10034259	C	A	-0.01616	0.002917	3.03E-08	0.198988
16	rs2389499	G	A	-0.01478	0.002701	4.44E-08	0.751887
17	rs187580	G	T	-0.01758	0.002763	1.96E-10	0.230751
18	rs9462364	G	A	0.0138	0.002332	3.26E-09	0.505824
19	rs2269426	A	G	0.018998	0.002415	3.62E-15	0.359398
20	rs3808072	T	C	0.016785	0.002651	2.41E-10	0.270741
21	rs274632	A	C	-0.01289	0.002342	3.75E-08	0.433318
22	rs10096972	C	T	-0.0184	0.003154	5.41E-09	0.834654
23	rs10959797	A	G	-0.01593	0.002854	2.39E-08	0.209896
24	rs28510415	G	A	0.022258	0.004022	3.13E-08	0.091617
25	rs4919695	G	A	-0.01742	0.002373	2.10E-13	0.395999
26	rs3026389	G	C	-0.01683	0.00284	3.13E-09	0.788882
27	rs10765762	T	C	-0.01406	0.002351	2.23E-09	0.541261
28	rs10750486	A	G	0.01308	0.002359	2.92E-08	0.501436
29	rs167915	T	A	-0.01434	0.002443	4.38E-09	0.352179
30	rs11067376	G	A	0.015104	0.002417	4.16E-10	0.359496
31	rs61957597	G	A	-0.01683	0.002755	9.96E-10	0.23255
32	rs7152906	C	T	0.014907	0.002318	1.28E-10	0.507194
33	rs55997507	C	G	0.015487	0.002406	1.22E-10	0.603649
34	rs17688916	A	T	0.01989	0.00299	2.87E-11	0.196725
35	rs58084604	T	C	-0.0168	0.002727	7.16E-10	0.236497
36	rs72893199	C	T	0.018261	0.002799	6.79E-11	0.221728
37	rs11152363	A	G	0.017198	0.003024	1.29E-08	0.184398
38	rs116962250	A	G	0.035277	0.006052	5.58E-09	0.038348
39	rs13054099	C	T	0.015024	0.00265	1.43E-08	0.257393

Table S11. Instrumental variables for "Irritableness".

No.	SNP	Effect allele	Other allele	Beta	Standard error	*p*-value	Eaf
1	rs7535528	A	G	0.013795	0.002456	1.93E-08	0.362619
2	rs4411173	A	C	0.018518	0.003168	5.08E-09	0.837493
3	rs343949	T	A	-0.01901	0.003104	9.09E-10	0.172046
4	rs11682175	C	T	0.013403	0.002351	1.20E-08	0.46613
5	rs6718682	T	C	-0.01589	0.00263	1.51E-09	0.274887
6	rs6711058	A	G	0.015985	0.002705	3.41E-09	0.733701
7	rs4953152	A	G	0.015411	0.002515	8.91E-10	0.315074
8	rs3774800	A	G	-0.01479	0.002443	1.40E-09	0.645971
9	rs3772556	T	C	-0.01447	0.002588	2.26E-08	0.712139
10	rs1542212	G	T	0.013514	0.002406	1.93E-08	0.39305
11	rs6549048	A	G	0.015419	0.002577	2.17E-09	0.292485
12	rs13157212	C	A	-0.01873	0.003432	4.84E-08	0.13417
13	rs6596771	A	G	-0.01399	0.002346	2.46E-09	0.486736
14	rs1422192	A	G	0.021833	0.003143	3.73E-12	0.165603
15	rs9403716	A	G	0.016292	0.002789	5.15E-09	0.227116
16	rs10228350	T	A	0.015521	0.002401	1.03E-10	0.412901
17	rs13223152	G	A	-0.01459	0.002397	1.15E-09	0.401671
18	rs3110417	G	T	0.015705	0.002703	6.21E-09	0.251491
19	rs4734804	G	A	-0.01572	0.002851	3.51E-08	0.220254
20	rs17151565	G	C	-0.01399	0.002421	7.59E-09	0.610732
21	rs1927903	C	G	-0.01349	0.002468	4.54E-08	0.658072
22	rs999483	G	T	0.019021	0.002706	2.07E-12	0.247877
23	rs10905638	C	G	0.014803	0.002358	3.41E-10	0.440857
24	rs102275	C	T	0.013874	0.00244	1.30E-08	0.35595
25	rs3026401	T	C	-0.01727	0.002853	1.42E-09	0.781595
26	rs7973253	G	A	0.014764	0.002427	1.18E-09	0.369666
27	rs3124405	T	G	-0.01691	0.002611	9.47E-11	0.722895
28	rs58446129	T	C	0.018108	0.00322	1.88E-08	0.156004
29	rs12886000	T	G	0.021126	0.003825	3.33E-08	0.105538
30	rs4781534	C	G	0.013721	0.002483	3.28E-08	0.343647
31	rs12931046	A	G	0.013959	0.002406	6.50E-09	0.615795
32	rs2106785	T	C	0.020883	0.002866	3.16E-13	0.213768
33	rs9630740	G	C	-0.0153	0.002658	8.65E-09	0.738335
34	rs2217127	G	T	-0.01416	0.002579	3.96E-08	0.709448
35	rs7231748	G	A	0.017429	0.002538	6.50E-12	0.306975
36	rs2587410	T	C	-0.01538	0.002688	1.05E-08	0.746939
37	rs62211616	A	G	0.019536	0.003046	1.43E-10	0.179217
38	rs4820434	T	G	0.014757	0.00254	6.27E-09	0.314394

Table S12. Instrumental variables for "Suffering from nerves".

No.	SNP	Effect allele	Other allele	Beta	Standard error	*p*-value	Eaf
1	rs10501320	C	G	6.85	0.998884	7.00E-12	0.25471
2	rs11665052	A	G	6.21	0.998536	5.00E-10	0.263176
3	rs12787112	A	G	8.13	0.998948	4.00E-16	0.340851
4	rs34467936	A	G	8.01	0.9979	1.00E-15	0.35405
5	rs35344466	A	C	6.37	1.001361	2.00E-10	0.221978
6	rs62081501	A	G	5.69	0.992893	1.00E-08	0.081337
7	rs6478623	T	G	-5.51	1.003487	4.00E-08	0.279275
8	rs7611991	A	G	-5.93	0.999731	3.00E-09	0.248899
9	rs9854869	A	C	-6.46	0.998925	1.00E-10	0.220189

Table S13. Instrumental variables for "Worrying after embarrassment".

No.	SNP	Effect allele	Other allele	Beta	Standard error	*p*-value	Eaf
1	rs1983614	T	C	0.012843	0.002335	3.79E-08	0.484257
2	rs9811585	G	T	0.017108	0.002402	1.06E-12	0.384784
3	rs793545	G	C	-0.01345	0.002447	3.87E-08	0.355248
4	rs6439649	T	G	0.015695	0.002387	4.82E-11	0.60374
5	rs362307	T	C	-0.02775	0.00453	9.05E-10	0.072192
6	rs35267052	G	T	0.021708	0.00387	2.03E-08	0.101497
7	rs3777095	A	G	-0.01334	0.002349	1.35E-08	0.46041
8	rs406204	T	C	-0.01576	0.00289	4.94E-08	0.206853
9	rs3999543	A	G	0.014568	0.00258	1.64E-08	0.305513
10	rs727903	T	A	0.014937	0.002346	1.94E-10	0.479377
11	rs72765272	C	A	-0.02127	0.003453	7.22E-10	0.1317
12	rs34588274	T	C	-0.01965	0.002382	1.60E-16	0.56477
13	rs2734837	T	C	-0.01442	0.002384	1.44E-09	0.594128
14	rs10750866	G	A	0.0179	0.00264	1.19E-11	0.272951
15	rs2160515	G	A	0.014739	0.002398	7.92E-10	0.429309
16	rs3742021	C	T	0.015749	0.002548	6.43E-10	0.302806
17	rs7987467	A	G	0.013359	0.00238	2.00E-08	0.407619
18	rs2191130	T	G	0.015113	0.002539	2.64E-09	0.678282
19	rs75022332	T	A	0.016093	0.002864	1.92E-08	0.213608
20	rs55731231	G	C	0.018613	0.002821	4.18E-11	0.221154
21	rs35327499	A	G	0.021872	0.003892	1.91E-08	0.102548
